# Clinical and molecular findings in three Japanese patients with *N*-acetylneuraminic acid synthetase-congenital disorder of glycosylation (NANS-CDG)

**DOI:** 10.1038/s41598-022-21751-x

**Published:** 2022-10-12

**Authors:** Yohei Masunaga, Gen Nishimura, Koji Takahashi, Tomiyuki Hishiyama, Masatoshi Imamura, Kenichi Kashimada, Machiko Kadoya, Yoshinao Wada, Nobuhiko Okamoto, Daiju Oba, Hirofumi Ohashi, Mitsuru Ikeno, Yuko Sakamoto, Maki Fukami, Hirotomo Saitsu, Tsutomu Ogata

**Affiliations:** 1 Department of Pediatrics, Hamamatsu University School of Medicine, Hamamatsu, Japan; 2 Center for Intractable Diseases, Saitama Medical University Hospital, Saitama, Japan; 3 Department of Pediatrics, Tsuchiura Kyodo General Hospital, Tsuchiura, Japan; 4 Department of Neonatology, Tsuchiura Kyodo General Hospital, Tsuchiura, Japan; 5 Department of Pediatrics and Developmental Biology, Tokyo Medical and Dental University, Tokyo, Japan; 6 Department of Molecular Medicine, Osaka Women’s and Children’s Hospital, Osaka, Japan; 7 Division of Medical Genetics, Saitama Children’s Medical Center, Saitama, Japan; 8 Department of Pediatrics, Juntendo University School of Medicine, Tokyo, Japan; 9 Department of Orthopedics, Juntendo University Nerima Hospital, Tokyo, Japan; 10 Department of Molecular Endocrinology, National Research Institute for Child Health and Development, Tokyo, Japan; 11 Department of Biochemistry, Hamamatsu University School of Medicine, Hamamatsu, Japan; 12 Department of Pediatrics, Hamamatsu Medical Center, Hamamatsu, Japan

**Keywords:** Genetics, Diseases, Endocrinology

## Abstract

We report clinical and molecular findings in three Japanese patients with *N*-acetylneuraminic acid synthetase-congenital disorder of glycosylation (NANS-CDG). Patient 1 exhibited a unique constellation of clinical features including marked hydrocephalus, spondyloepimetaphyseal dysplasia (SEMD), and thrombocytopenia which is comparable to that of an infant reported by Faye-Peterson et al., whereas patients 2 and 3 showed Camera-Genevieve type SMED with intellectual/developmental disability which is currently known as the sole disease name for NANS-CDG. Molecular studies revealed a maternally inherited likely pathogenic c.207del:p.(Arg69Serfs*57) variant and a paternally derived likely pathogenic c.979_981dup:p.(Ile327dup) variant in patient 1, a homozygous likely pathogenic c.979_981dup:p.(Ile327dup) variant caused by maternal segmental isodisomy involving *NANS* in patient 2, and a paternally inherited pathogenic c.133−12T>A variant leading to aberrant splicing and a maternally inherited likely pathogenic c.607T>C:p.(Tyr203His) variant in patient 3 (reference mRNA: NM_018946.4). The results, together with previously reported data, imply that (1) *NANS* plays an important role in postnatal growth and fetal brain development; (2) SMED is recognizable at birth and shows remarkable postnatal evolution; (3) NANS-CDG is associated with low-normal serum sialic acid, obviously elevated urine *N*-acetylmannosamine, and normal *N*- and *O*-glycosylation of serum proteins; and (4) NANS-CDG is divided into Camera-Genevieve type and more severe Faye-Peterson type.

## Introduction

*N*-acetylneuraminic acid synthetase-congenital disorder of glycosylation (NANS-CDG) is a recently established rare autosomal recessive disease caused by pathogenic variants in *NANS* involved in the biosynthesis of N-acetylneuraminic acid (the most common member of sialic acids)^1,2^. Sialic acids occupy the terminal position of sugar chains of glycoproteins and glycolipids including gangliosides, and participate in a variety of physiological processes such as interaction between various cells, recognition of pathogens, and stabilization of proteins^3^. Sialic acids are ubiquitously distributed in the body including the brain and skeletal system^4–7^, and are required for the development and function of multiple organs/tissues^3^. Consistent with this, NANS-CDG leads to Camera-Genevieve type spondyloepimetaphyseal dysplasia (SEMD) (OMIM **#**610442) associated with infantile-onset intellectual developmental disability (IDD) as well as other various clinical features^1,2^.

Here, we report clinical and molecular findings in three Japanese patients with NANS-CDG. The results, in conjunction with the previous data, imply that biallelic *NANS* variants cause not only Camera-Genevieve type SMED but also more severe phenotype including marked hydrocephalus as documented in an infant reported by Faye-Peterson et al.^8^.

## Results

### Clinical description

Clinical features of patients 1–3 are summarized in Table 1. Patients 1–3 were conceived naturally. Patient 1 was noticed to have marked ventriculomegaly by echography at 20 weeks of gestation and severe hydrocephalus with cortical thinning by magnetic resonance imaging (MRI) at 32 weeks of gestation. Patient 2 was found to have mild ventriculomegaly by fetal echography at 32 weeks of gestation. Patient 3 was noticed to have shortened femoral length by repeated fetal echography. Patient 1 was delivered by Caesarean section at 35 weeks of gestation because of progressive head enlargement, whereas patients 2 and 3 were born at term via vaginal delivery. After birth, patient 1 exhibited intractable respiratory distress because of thoracic dysplasia and recurrent aspiration pneumonia, and was placed on oxygen therapy and nasogastric tube feeding. He also received ventriculoperitoneal shunt at one month of age, tracheotomy at 5 months of age, and gastrostomy at 6 months of age. Patients 2 and 3 experienced no such severe clinical episode.

**Table 1:** Clinical findings in patients with NANS-CDG.

	Patient 1	Patient 2	Patient 3	Reported cases (n = 17)*
Present/Reported age (y:m)	1:10	9:3	7:8	0–40
Gender	Male	Female	Male	M 7, F 10
**Pregnancy and delivery**				
Conception	Natural	Natural	Natural	Natural 17/17
Prenatal abnormality	+	+	+	3/17
Gestational age (w)	35	40	40	35–41
Delivery	Caesarean	Vaginal	Vaginal	Caesarean 2/17
**Growth pattern**				
Prenatal growth failure^a^	+	+	+	3/9
Birth length cm (SD)	40.5 (− 2.0)	45.0 (− 2.7)	45.1 (− 2.4)	P < 0.4–50
Birth weight kg (SD)	2.30 (− 0.1)	2.97 (− 0.4)	2.65 (− 1.4)	P < 2.5–97
Birth head circumference cm (SD)	39.0 (+ 5.3)	33.5 (− 0.1)	32.0 (− 1.1)	P < 2.5–98
Postnatal growth failure^a^	+	+	+	16/17
Age at examination (y:m)	1:6	8:11	7:4	
Length/height cm (SD)	64.0 (− 6.4)	95.0 (− 6.0)	103.5 (− 3.5)	P < 0.01–44^d^
Weight kg (SD)	9.3 (−1.5)	12.8 (− 2.7)	15.9 (− 1.9)	P < 0.01–95^d^
Head circumference cm (SD)	53.0 (+ 4.2)	46.0 (− 4.4)	48.3 (− 2.5)	P < 0.01– > 95
Arm span cm (SD)	51.0 (…)^b^	93.0 (− 4.9)	108.0 (− 1.8)	…
Arm span/height ratio (SD)	0.80^b^	0.98 (± 0.0)	1.04 (+ 2.4)	…
**Developmental status**				
Intellectual developmental disability	+	+	+	17/17
Motor delay	+	+	+	15/15
Speech delay	Unknown^c^	+	+	16/16
Seizures	–	–	–	5/17
**Physical findings**				
Facial dysmorphisms	+	+	+	15/17
Coarse facies	+	+	+	5/5
Prominent forehead	+	–	–	7/11
Ptosis	+	+	+	2/2
Sunken nasal bridge	+	+	+	13/13
Full lips	+	+	+	4/4
Strabismus	+	–	+	8/12
Dental misalignment	–	+	–	3/3
Disproportionate short limbs	+	–	+	13/17
Scoliosis	+	+	+	4/4
Other features	Footnote-1	…	Footnote-3	
**Skeletal roentgenographic findings**				
Spondyloepimetaphyseal dysplasia	+	+	+	14/17
**Brain MRI**				
Age at examination (y:m)	0:2	1:0	0:9	
Ventricular dilatation	+ (Severe)	+	+	10/10
Septum pellucidum abnormalities	+	+	+	8/17
Periventricular white matter lesion	–	–	–	2/2
Other findings	Footnote-2	Unknown	Unknown	
**Laboratory findings**				
Age at examination (y:m)	0:7	4:4	6:1	
Thrombocytopenia	+	–	+	5/17
Megathrombocytes	+	…	+	…
Platelet (10^4^/µL)^†^	0.6	24.0	3.5	2.9–19.2
Immature platelet fraction (%)^†^	37.6	3.4	…	…
Platelet associated IgG (ng/10^7^cells)^†^	342	…	…	…
Age at examination (y:m)	1:4	8:11	6:9	
Serum total sialic acid (mg/dL)^†^^**‡**^	48	49	50	…
Age at examination (y:m)	1:10	9:2	7:7	
Urine ManNAc (µmol/mmol cr)^†^	330.0	76.5	37.6	10–530
**Parental information**				
Paternal height cm (SD)	170.5 (− 0.1)	165 (− 1.0)	174 (+ 0.6)	…
Maternal height cm (SD)	160.8 (+ 0.5)	155 (− 0.6)	164 (+ 1.1)	…
Paternal age at childbirth (y)	30	32	32	…
Maternal age at childbirth (y)	25	38	31	…
Paternal urine ManNAc (µmol/mmol cr)^†^	1.46	1.85	…	…
Maternal urine ManNAc (µmol/mmol cr)^†^	3.96	1.95	2.12	…

y, year; m, month, w, week, SD, standard deviation; MRI, magnetic resonance imaging; ManNAc, N-acetylmannosamine, cr, creatinine; and P, percentile.

*van Karnebeek et al.^1^ and den Hollander et al.^2^.

^†^Reference values: 15.0–35.0 for platelet, 1.1–6.1 for immature platelet fraction, < 46 for platelet associated IgG, 44–71 for sialic acids, and 0.71–3.99 for urine ManNAc; the reference value for ManNAc is derived from the data in 10 control subjects examined in this study (five children and five adults with no obvious difference between the children and the adults), and the remaining reference vales are based on the data of Special Reference Laboratories, Inc., Japan.

^‡^Free plus glycoprotein-bound and glycolipid-bound sialic acid.

For the frequency in reported cases, the denominators indicate the number of patients examined for the presence or absence of each feature, and the numerators represent the number of patients assessed to be positive for that feature.

^a^Birth or present length/height ≤ − 2 SD or P 3.

^b^No reference data for Japanese children ≤ 6 years.

^c^Unknown because of post-tracheotomy.

^d^A single patient (patient 8 in Hollander et al.^2^) alone exhibits normal stature (P 44) and large weight (P 95).

Footnote-1: External auditory canal stenosis, auditory disturbance, bulbous nasal tip, megaloglossia, short neck, and thoracic dysplasia.

Footnote-2: Thinning of cortex and white matter, and narrowed cerebral aqueduct.

Footnote-3: Intermittent exotropia, hyperopia, brachycephaly, hypertelorism, anteverted nares, short philtrum, protruding ears, and cup shaped ears.

Growth pattern analysis revealed mildly decreased birth lengths and low-normal birth weights, and severely compromised postnatal length/heights in patients 1–3, especially in patients 1 and 2, and mildly decreased or low-normal postnatal weights in patients 1–3. Patient 1 had markedly enlarged head circumference (HC) since birth, whereas patients 2 and 3 had low-normal birth HCs and severely or mildly decreased postnatal HCs. Arm span was severely reduced with a normal arm span to height ratio in patient 2, whereas it remained at a low-normal range with a mildly increased arm span to height ratio in patient 3.

IDD was obvious in patients 1–3. Patient 1 was unable to control his head at 1 6/12 years of age. Patient 2 was incapable of rolling until three years of age, sitting until 4 years of age, and standing with support until 5 years of age, and she was just able to speak single words at 8 11/12 years of age. Patient 3 was incapable of controlling his head until 8 months of age, sitting until 2 2/12 years of age, and walking until 5 5/12 years of age, and he was still unable to speak single words at 7 4/12 years of age.

**Figure 1:**
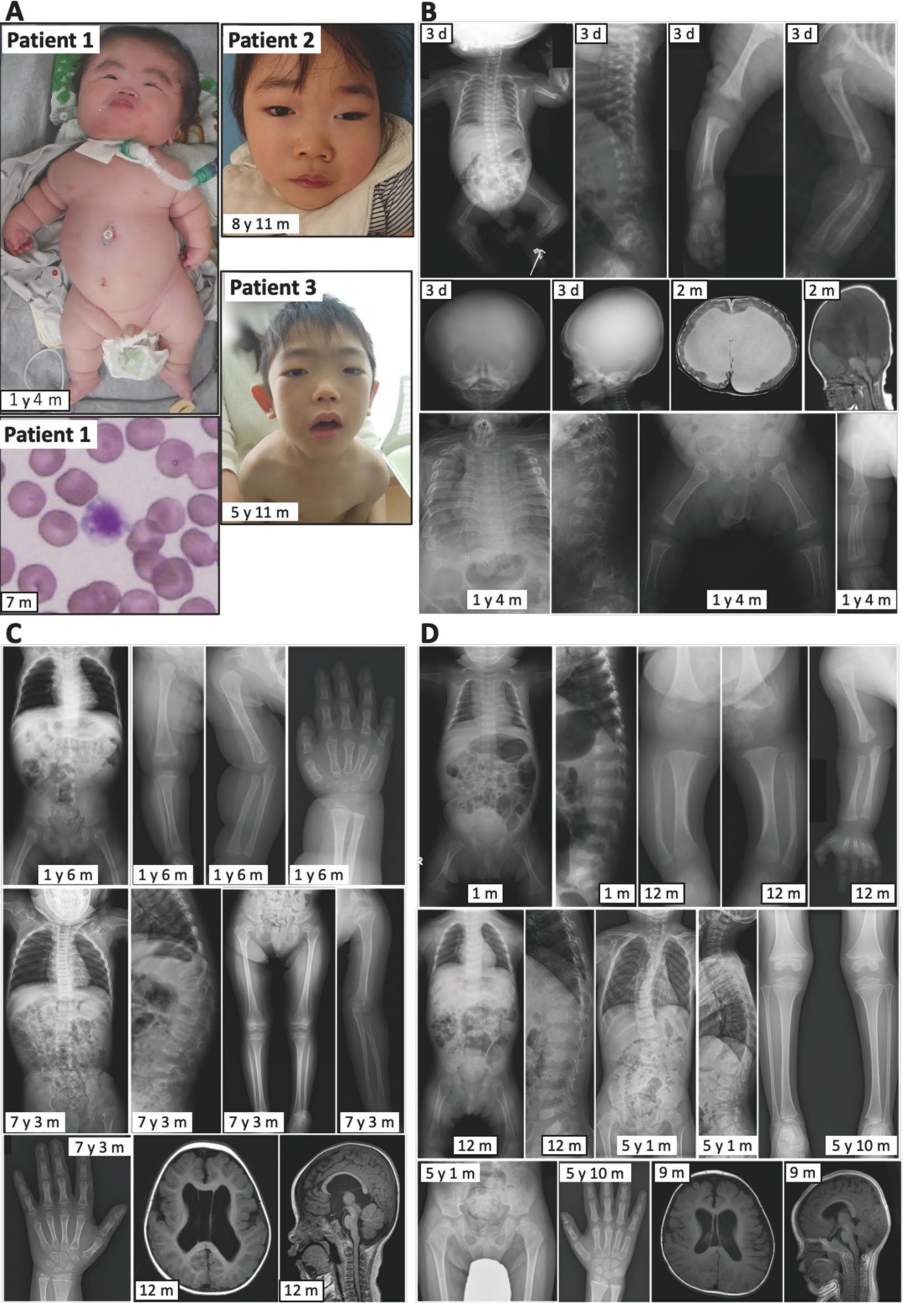
Clinical findings in patients 1–3. y, year; m, month; and d, day. (**A**) Photographs of patients 1–3 and blood smear of patient 1 showing a megathrombocyte. (**B–D**) Radiological findings in patients 1–3, respectively.

Physical examination was repeatedly performed before and after the identifications of *NANS* variants. Consequently, patients 1–3 were found to have various features previously reported in NANS-CDG, including characteristic facial appearance (Fig. 1A).

Roentgenographic examinations were also repeatedly performed, revealing SEMD with remarkable age-dependent evolution (Fig. 1B–D). Skeletal hallmarks in the neonatal period included a mildly narrow thorax, mild platyspondyly (flat vertebral bodies) with multiple coronal clefts, broad ilia with short greater sciatic notches and horizontal acetabula, and stubby long bones. In childhood, platyspondyly became more conspicuous along with development of vertebral endplate irregularities. In addition, epiphyseal ossification of the long bones was irregular and flocky in appearance, and carpal ossification was advanced. The long bones became overtubulated together with metaphyseal flaring and subphyseal longitudinal striations with age in patients 2 and 3 (Supplementary Fig. 1).

Brain MRI delineated marked hydrocephalus with callosal atrophy and stenosis of the cerebral aqueduct in patient 1, and modest lateral ventriculomegaly in patients 2 and 3. Patients 1–3 had septum pellucidum abnormalities, but not periventricular white matter lesion (Fig. 1B–D).

Laboratory studies revealed persistent thrombocytopenia since infancy, in association with the appearance of megathrombocyte in the peripheral blood of patients 1 and 3. In patient 1, immature platelet fraction and platelet-associated IgG (PAIgG) were elevated and, although the platelet count increased from 0.6 to 11.0 × 10^4^/µL after platelet transfusion, it decreased from 11.0 to 1.4 × 10^4^/µL in six days. This decrease rate was apparently faster than a standard half-life time of circulating platelet of 3–5 days. In patient 3, bone marrow examination showed atypia of three kinds of hematopoietic cells, i.e. abnormal granules in neutrophils, karyolysis in erythroblasts, and micro megakaryocytes. Although patient 3 was treated with oral prednisolone, cyclosporin A, and eltrombopag (a stimulator of thrombopoietin receptor), these drugs were ineffective for thrombocytopenia. Furthermore, blood total (free plus glycoprotein-bound and glycolipid-bound) sialic acid values measured after the identification of *NANS* variants were at a low-normal range in patients 1–3, whereas urine *N*-acetylmannosamine (ManNAc) (the substrate for NANS) values were obviously increased in patients 1–3, especially in patient 1. Endocrine studies for short stature including serum IGF-I value and thyroid function revealed no abnormality in patients 1–3.

Thus, the overall phenotype of patient 1, including the severe hydrocephalus and markedly enlarged HC, appeared atypical for Camera-Genevieve type SMED, whereas that of patients 2 and 3 was consistent with Camera-Genevieve type SMED^1,2^.

The parents of patients 1–3 were non-consanguineous and healthy, with normal heights. The childbearing age was advanced in the mother of patient 2. Urine ManNAc values were within the normal range in all the five parents examined.

### Molecular studies

**Figure 2:**
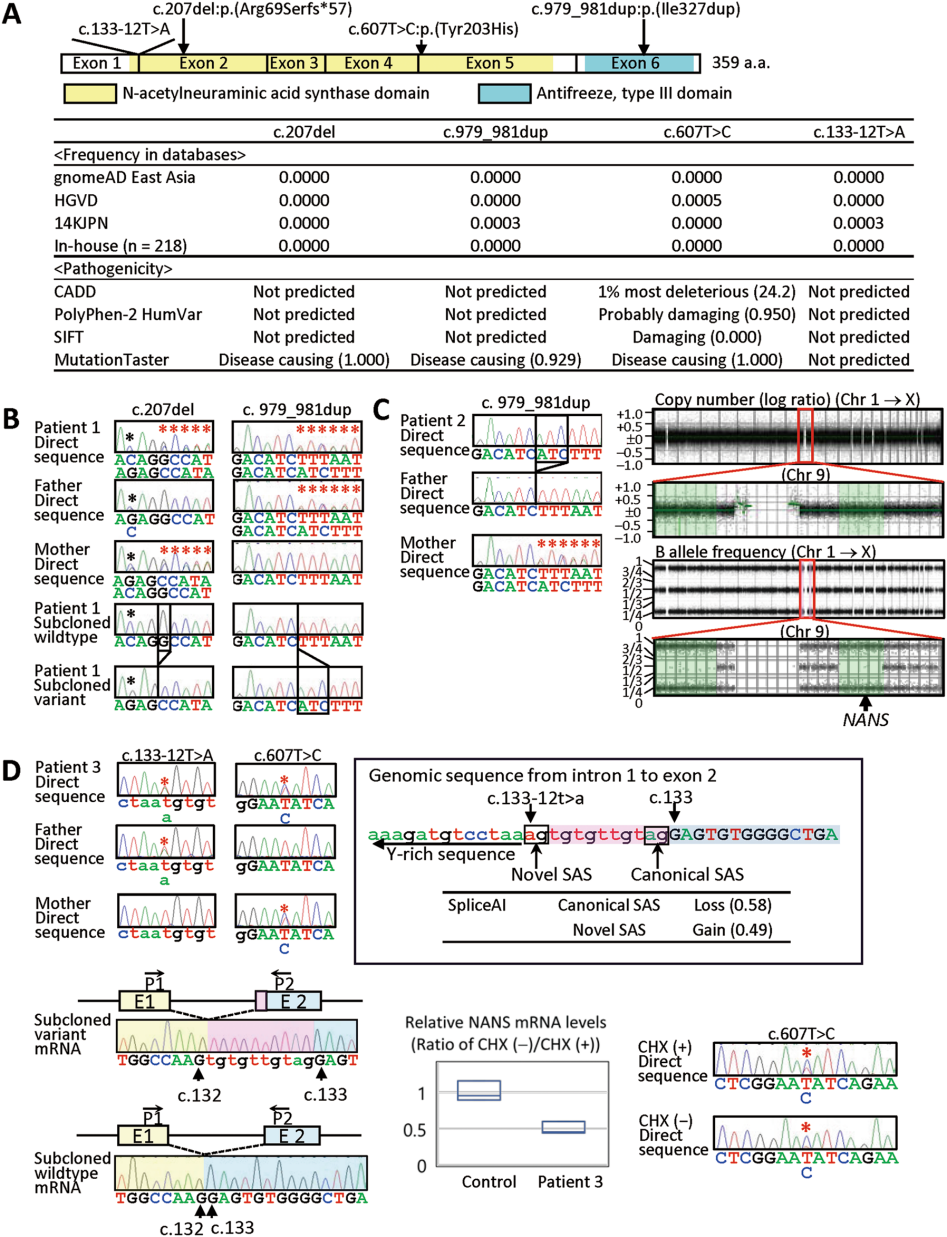
Molecular findings in patients 1–3. (**A**) The position and the frequency and pathogenicity of the *NANS* variants identified in this study (see also Supplementary Tables 1–3). (**B**) Molecular data in patient 1, showing the c.207del and c.979_981dup variants. Red asterisks indicate the frameshifted direct sequences. Black asterisks denote a G/C SNP (rs1058446; allele frequency, G 81% and C 19%). (**C**) Molecular data in patient 2, showing the homozygous and heterozygous c.979_981dup variant in patient 2 and the mother, respectively. Red asterisks indicate the frameshifted direct sequences. CytoScan HD analysis reveals the normal copy number and segmental loss of heterozygosity for chromosome 9, indicating segmental isodisomic regions involving *NANS* (highlighted with light green). (**D**) Molecular data in patient 3, showing the c.133–12T>A and c.607T>C variants (indicated with red asterisks). The c.133–12T>A creates a novel splice acceptor site (SAS) which is predicted to be utilized more preferentially than the canonical SAS by SpliceAI and produce an aberrant mRNA subject to NMD. Consistent with this, experiments using mRNA samples reveal a variant mRNA containing 10 bp intronic sequence on the electrochromatogram for subcloned mRNAs, a roughly halved mRNA expression ratio between CHX-untreated and CHX-treated LCLs, and a reduced wildtype "T" peak for the c.607T>C variant on the electrochromatogram obtained by direct sequencing for the CHX untreated LCLs (indicated with red asterisks).

We performed whole exome sequencing, using leukocyte genomic DNA samples of patients 1–3 and their parents. Consequently, we identified following extremely rare *NANS* variants: (1) a maternally inherited c.207del:p.(Arg69Serfs*57) variant and a paternally derived c.979_981dup:p.(Ile327dup) variant in patient 1; (2) a homozygous or hemizygous c.979_981dup:p.(Ile327dup) variant in patient 2 and a heterozygous c.979_981dup:p.(Ile327dup) variant in the mother; and (3) a c.607T>C:p.(Tyr203His) variant of maternal origin and a c.133−12T>A variant of paternal origin in patient 3 (reference mRNA: NM_018946.4) (Fig. 2A). These variants were confirmed by Sanger direct sequencing in patients 1–3 and their parents and by sequencing of subcloned wildtype and variant alleles in patient 1 (Fig. 2B–D). The c.979_981dup:p.(Ile327dup) variant was previously reported in two siblings with NANS-CDG^1^, whereas the remaining variants were novel. While several rare (minor allele frequencies of ≤ 0.01) variants were also revealed in other genes of patients 1–3, the overall assessment based on in silico pathogenicity predictions and phenotypic information documented in OMIM appeared to argue against their relevance to clinical findings in patients 1–3 (Supplementary Tables 1–3).

Thus, we assessed the *NANS* variants using the ACMG guideline^9^. In patient 1, the p.(Arg69Serfs*57) variant was evaluated as likely pathogenic, because it was positive for PVS1 (a null variant) and PM2 (absent from controls). The p.(Ile327dup) variant was also assessed as likely pathogenic, because it was positive for PM2 (extremely low frequency in a recessive disorder), PM3 (detected in *trans* with a null variant for a recessive disorder), PM4 (protein length changes in a nonrepeat region), and PP5 (a previously reported variant without functional evidence).

In patient 2, we performed CytoScan HD analysis, to examine homozygosity or hemizygosity for the likely pathogenic p.(Ile327dup) variant. Consequently, segmental isodisomic regions associated with normal copy number and loss of heterozygosity were identified on chromosome 9, with the centromeric portion being heterodisomic (Fig. 2C). Since *NANS* was present on the isodisomic region, this showed homozygosity for the likely pathogenic variant in patient 2.

In patient 3, we examined the pathogenicity of the c.133−12T>A variant (Fig. 2D). This variant created the ″ag″ consensus splice acceptor motif, and SpliceAI^10^ predicted splice loss for the canonical splice acceptor site and splice gain for the novel splice acceptor site. Thus, this variant was predicted to produce an aberrant mRNA containing 10 intronic sequence subject to nonsense-mediate mRNA decay (NMD)^11^. To confirm this, we carried out reverse transcription (RT)-PCR for mRNA extracted from lymphoblastoid cell lines (LCLs) cultured for 8 h with and without NMD-inhibitor cycloheximide (CHX), using primers hybridizing to exons 1 and 2. Subsequently, Sanger sequencing was performed for subcloned mRNA, revealing the aberrant mRNA containing 10 intronic nucleotides in CHX-treated LCLs, together with wildtype mRNA. Quantitative RT-PCR was also carried out with the same primers, indicating that relative *NANS* mRNA quantity between CHX-untreated and CHX-treated LCLs was nearly halved in patient 3, as compared with that of a control subject. Furthermore, Sanger sequencing for the c.607T>C:p.(Tyr203His) variant using CHX-untreated and CHX-treated LCLs indicated that the paternally inherited allele with c.133−12T>A variant and the wildtype c.607T predominantly underwent NMD. Thus, the c.133−12T>A was described as r.132_133insTGTGTTGTAG:p.(Glu45Cysfs*8) and was assessed as pathogenic, because it was positive for PSV1 (functionally null variant), PM2 (see above), and PP4 (phenotype highly specific for the variant). Accordingly, the p.(Tyr203His) variant was evaluated as likely pathogenic, because it was positive for PM2 (see above), PM3 (see above), and PP4 (see above).

### Glycosylation studies

**Figure 3:**
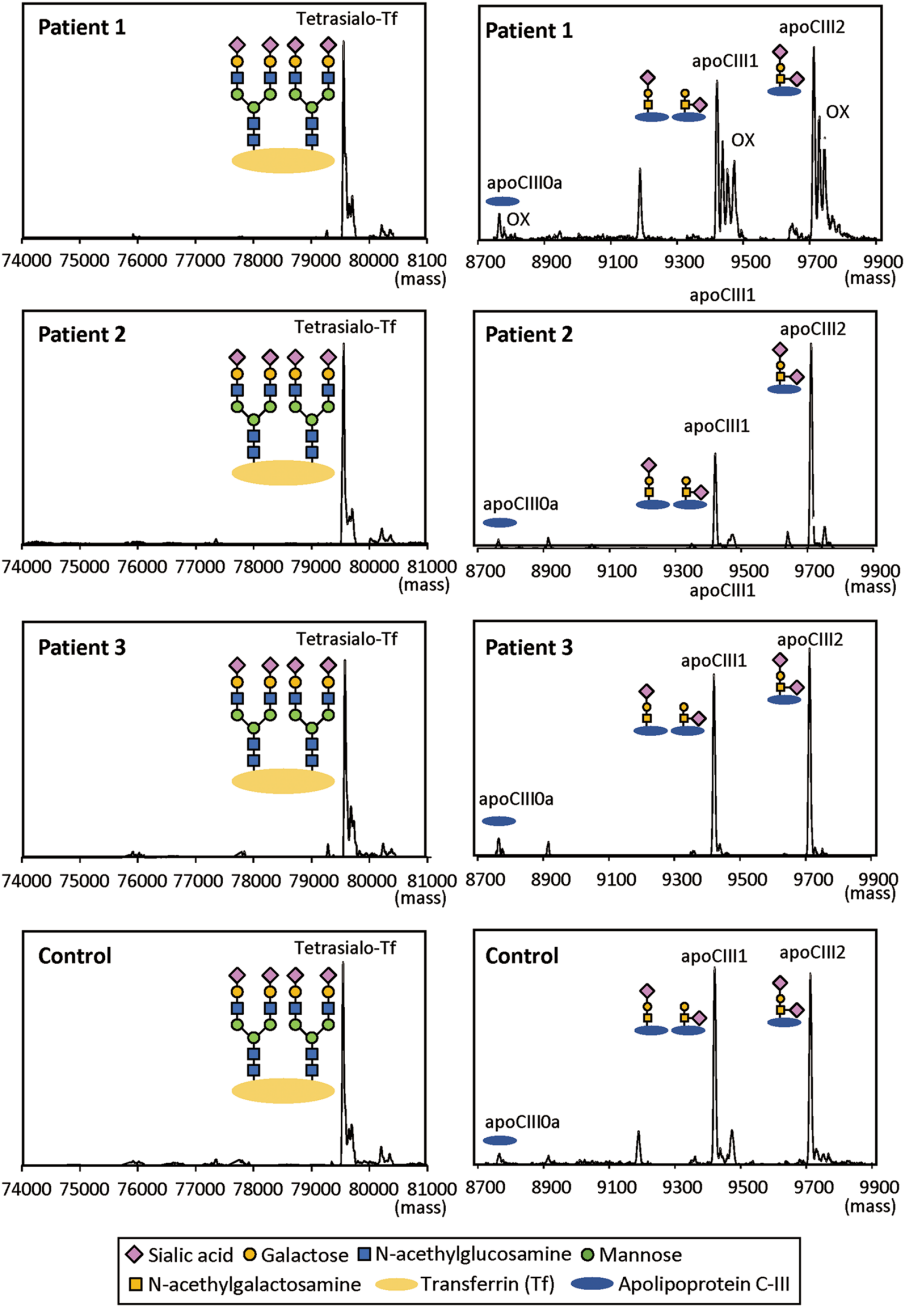
Glycosylation status of serum proteins in patients 1–3 and a control subject. The *N*-glycosylation status of transferrin (left) and the *O*-glycosylation status of apolipoprotein C-III (right) are normal in patients 1–3. Since the *O*-glycosylation status of apolipoprotein C-III in patient 1 was examined using serum sample stored at − 20 °C for 6 months, additional peaks due to oxidation of the core protein backbone (OX) are delineated. Other measurements were performed using fresh serum samples.

We examined the *N*-glycosylation status of transferrin and the O-glycosylation status of apolipoprotein C-III by mass spectrometry. Despite the presence of *NANS* variants, both glycosylation patterns were normal in patients 1–3 (Fig. 3).

## Discussion

Whole exome sequencing identified biallelic pathogenic or likely pathogenic variants in *NANS* of patients 1–3. Furthermore, no other pathogenic variant which could explain the clinical features of patients 1–3 was detected. The results imply that patients 1–3 have NANS-CDG. In this regard, one may pose a question regarding the pathogenicity of the p.(Ile327dup) variant associated with just one amino acid duplication. However, it would be regarded as disease-causing, because patient 2 with homozygosity for the p.(Ile327dup) variant showed Camera-Genevieve type SEMD-compatible phenotype and the elevated urine ManNAc value which is regarded as pathognomonic for NANS-CDG^2^. In this regard, the p.(Ile327dup) variant has previously been identified in two Japanese siblings with NANS-CDG, although functional studies have not been performed^1^, and it has been registered with a frequency of 0.0003 in the Japanese database (14KJPN) (Fig. 2A). Thus, the p.(Ile327dup) variant might be prevalent or specific in the Japanese population.

Notably, while patient 1 had a simple compound heterozygosity for the variants on the coding sequence, patients 2 and 3 had unique molecular findings. Indeed, patient 2 had segmental 9q isodisomy which led to unmasking of the maternally inherited *NANS* variant. Since the centromeric portion was heterodisomic, this implies that patient 2 had upd(9)mat generated by premature sister chromatid separation or non-disjunction at maternal meiosis 1 followed by trisomy rescue, consistent with the advanced maternal childbearing age^12^. Similarly, patient 3 had an intronic variant which was preferentially utilized as a novel splice acceptor site. Since Sanger sequencing for CHX-untreated LCLs delineated a low but discernible peak for the wildtype c.607T, this may suggest that a small amount of mRNA generated by the novel splice acceptor site escaped NMD or that a small amount of mRNA was produced by the canonical rather than the novel splice acceptor site on the paternally derived chromosome.

Patients 1–3 showed salient clinical features in NANS-CDG such as growth failure, brain anomalies with IDD, and SMED^1,2^. In this regard, several findings are notable. First, growth failure became severe after birth, as has been described previously^1,2^. This would imply that *NANS* plays an important role in the postnatal rather than prenatal growth. Second, ventriculomegaly was noticed during the fetal life in patients 1 and 2. This would suggest that defective brain development takes place in the fetal life. In particular, patient 1 had severe hydrocephalus and markedly enlarged HC, in association with stenosis of the cerebral aqueduct. In this regard, rat experiments have shown that neuraminidase injection into the lateral ventricle results in the cleavage of sialic acids from ependymal surface glycoproteins or glycolipids and in the fusion of the lateral walls of cerebral aqueduct^13^. Thus, it is likely that the hyposialylated condition in the ependyma has caused the stenosis of the cerebral aqueduct, leading to the severe hydrocephalus and markedly enlarged HC in patient 1. Third, NANS-CDG skeletal features were recognizable at birth, and showed remarkable postnatal evolution. In the neonatal period, the combination of modest platyspondyly with multiple coronal clefts of the spine would represent a diagnostic clue for NANS-CDG, whereas in childhood coronal clefts diminish over early childhood and, instead, subphyseal longitudinal striations, advanced carpal ossification, and irregular and sclerotic epiphyses (flocky epiphyses) would serve as the key skeletal features for NANS-CDG.

Several findings are also worth pointing out. First, thrombocytopenia was observed in patients 1 and 3. This would be due to hyposialylation status, because: (1) patients with disease-causing variants in *GNE* or *SLC35A1* involved in a sialylation pathway also often exhibit thrombocytopenia, in association with an exaggerated platelet clearance in the liver^14–16^; and (2) removal of sialic acids from platelets by neuraminidase treatment has resulted in shortened platelet life, in association with elevated serum PAIgG levels in baboons^17^. Second, serum total (free plus glycoprotein-bound and glycolipid-bound) sialic acid values remained at a low-normal range, whereas urine ManNAc values were unequivocally elevated in patients 1–3. Previous studies have also revealed normal sialic acid values in the cerebrospinal fluid and urine (no serum data available)^1^ and increased blood and urine ManNAc values in NANS-CDG^1,2^. These findings imply that increased ManNAc represents the hallmark for the diagnosis of NANS-CDG^2^. For the normal sialic acid values, it is inferred that at least one *NANS* variant retains some residual activity, producing a certain amount of sialic acids using the elevated ManNAc. Indeed, no patient with apparently biallelic amorphic variants has been described^1,2^, and *Gne* knockout mice deficient for sialic acids are embryonic lethal^18^. In addition, salvage pathway-derived and nutrition-derived sialic acids may also have contributed to the low-normal serum sialic acid values. Furthermore, the low-normal serum sialic acid values, in conjunction with the previous data suggesting a limited effect of orally administrated sialic acids^19^, may suggest that decreased local, rather than circulating, sialic acids play a critical role in the phenotypic development in NANS-CDG. Third, the *N*- and *O*-glycosylation statuses of serum proteins were normal, as reported previously^1^. This would be consistent with the low-normal serum sialic acid values.

Clinical findings are obviously more severe in patient 1 than in patients 2 and 3. Indeed, while phenotypes of patients 2 and 3 are consistent with Camera-Genevieve type SEMD, patient 1 had markedly enlarged HC as compared with his birth length and severe thrombocytopenia, in addition to marked postnatal growth failure, IDD, and SEMD. This would be due to the variable expressivity rather than genetic heterogeneity in NANS-CDG, because patient 1 had *NANS* variants as the sole identified pathogenic variants which could explain his clinical findings including the elevated urine ManNAc value. Indeed, since urine ManNAc value was much more elevated in patient 1 than in patients 2 and 3, this suggests that the residual NANS activity is much lower in patient 1 than in patients 2 and 3, and explains the phenotypic difference between patient 1 and patients 2 and 3. Such a correlation between urine ManNAc value and clinical severity has been reported previously^2^. To our knowledge, Faye-Peterson et al. have reported an infant with clinical features similar to those of patient 1, such as growth failure with a birth length of < 5th percentile, marked hydrocephalus with a birth HC of > 97th percentile, agenesis of the corpus callosum, SEMD, and thrombocytopenia, although molecular studies have not been performed^8^. Furthermore, van Karnebeek et al. have reported a *NANS*-variant positive patient (patient 8) with a marked disproportion between birth HC (98th percentile) and a birth length (< 0.4th percentile), although this patient lacked thrombocytopenia^1^, and den Hollander et al. have also described a *NANS*-variant positive patient (patient 3) with an obvious disproportion between birth HC (82th percentile) and birth length (2nd percentile) and thrombocytopenia^2^. These findings suggest that NANS-CDG is classified into Camera-Genevieve type and more severe Faye-Peterson type.

In summary, we observed three Japanese patients with NANS-CDG. The results, together with the previous data, imply that NANS-CDG is caused by various *NANS* variants and is associated with a wide phenotypic spectrum including Camera-Genevieve type and more severe Faye-Peterson type. Further studies will permit to reveal more precise molecular and phenotypic spectrum of NANS-CDG.

## Methods

### Ethical approval

This study was approved by the Institutional Review Board Committee of Hamamatsu University School of Medicine, and all experimental methods were performed in accordance with guidelines and regulations of Hamamatsu University School of Medicine. We obtained written informed consent for study participation and for the publication of patient’s clinical information including photos from the parents of patients 1–3.

### Primers

Primers utilized in this study are shown in Supplementary Table 4.

### Molecular studies using leukocyte genomic DNA samples

Whole exome sequencing was carried out using SureSelect Human All Exon V6 (Agilent Technologies). Captured libraries were sequenced by NextSeq 500 (Illumina) with 150 bp paired-end reads, and reads were aligned to the reference genome (Human GRCh38) (http://genome.ucsc.edu/), using BWA-MEM (Version 0.7.12) with default parameters. Duplicated reads were removed by Picard (Version 2.9.2), and local realignment and base quality recalibration were performed by GATK Version 3.7. Variants were identified with the GATK HaplotypeCaller. We extracted rare variants with minor allele frequencies of ≤ 0.01 in all the databases utilized in this study, and performed in silico pathogenicity predictions for extracted rare variants. The databases and in silico pathogenicity prediction methods are described in Supplementary Table 1, together with their URLs.

Sanger direct sequencing was performed for PCR products containing identified variants on the ABI 3130xl Genetic Analyzer (ThermoFisher Scientific). To confirm the variant, the PCR products were subcloned with the TOPO TA Cloning Kit (ThermoFisher Scientific), and wildtype and variant alleles were sequenced separately.

Copy number variant (CNV) and B-allele frequency (BAF) analyses were performed using CytoScan HD (ThermoFisher Scientific) harboring more than 2.4 million CNV markers and approximately 750,000 SNP markers. The log ratio and BAF were calculated using Rawcopy R package^20^. We selected CNVs larger than 100-kb, using log-ratio thresholds of >  + 0.2 for gains and < − 0.3 for deletions, and these CNVs were annotated using AnnotSV (Annotation and Ranking of Human Structural Variations) (https://www.lbgi.fr/AnnotSV/runjob). We searched for known pathogenic CNVs and CNVs of unknown significance absent from multiple public databases adopted in AnnotSV.

### Molecular studies using LCL-derived mRNA

Total RNA samples were isolated from immortalized LCLs cultured in media with or without CHX (Sigma) for 8 h, using RNeasy Mini Kit (QIAGEN). RT-PCR was performed for one μg of total RNA, using ReverTra Ace qPCR RT Kit (TOYOBO). The RT-PCR products were subjected to direct sequencing and quantitative RT-PCR by the SYBR Green methods on StepOnePlus system with Software v2.2.2 (ThermoFisher Scientific), using *ATCB* as internal controls.

### Serum sialic acid measurement

Total (free plus glycoprotein-bound and glycolipid-bound) serum sialic acids were enzymatically assayed by the previously reported method^21^, with minor modifications. In brief, glycoprotein-bound and glycolipid-bound sialic acids were hydrolyzed to free form by neuraminidase. Then, the total sialic acids (originally free sialic acids plus originally glycoprotein-bound and glycolipid-bound sialic acids) were cleaved to ManNAc and pyruvic acid by N-acetyl neuraminic acid-aldolase, and pyruvate was measured by means of LDH with NADH as a change of absorbance at 340 nm. Sialic acid value was determined with N-acetylneuranimic acid as a standard.

### Urine ManNAc measurement

Urine ManNAc was measured by hydrophilic interaction liquid chromatography mass spectrometry using a SeQuant ZIC-HILIC column (1 mm diameter and 150 mm length, Merck) connected to an API4500 mass spectrometer. The two mobile phases used were 0.2% acetic acid (AA) and 0.05% trifluoroacetic acid (TFA) (solvent A), and 0.2% AA and 0.05% TFA in acetonitrile (solvent B)^22^. After removing the precipitate by centrifugation, the urine was diluted 1/100 with solvent B, and a 20 μL portion was injected onto the column. The following gradient was applied at a flow rate of 0.065 mL/min; linearly decreased from 97 to 50% of solvent B from 0 to 40 min, further decreased to 30% B from 40 to 45 min. The analytes were monitored using selective reaction monitoring in positive-ion electrospray ionization mode. The collision energy was set at 15 eV, and the transitions monitored were m/z 222 → 126 and 222 → 138. The latter was used for discrimination of ManNAc from *N*-aetylglucosamine, and N-acetylgalactosamine was eluted earlier than ManNAc. A linear response was obtained from 0.5 to 250 pmol of ManNAc, and the sample amounts described above were within this range. Stable isotope-labeled internal standards were not required for urine samples, as the sample preparation was very simple and did not involve an extraction step. The coefficient of variation was 1.9%.

### Glycosylation studies

The *N*-glycosylation status of transferrin and the O-glycosylation status of apolipoprotein C-III were examined by electrospray ionization (ESI) MS as described previously^23,24^. In brief, each protein was purified from serum by immunoaffinity with rabbit polyclonal antibody against human transferrin (Dako) or goat polyclonal antibody against human apolipoprotein C-III (Academy Bio-Medical Co), and was subjected to liquid chromatography-ESI–MS using a reversed-phase, C4 for transferrin and C8 for apolipoprotein C-III, minicolumn and an API4500 quadrupole mass spectrometer (Sciex). The obtained mass spectrum of multiply-charged ions was transformed into a single-charge spectrum using a Promass protein deconvolution software (ThermoFisher Scientific).

## Supplementary Information

Supplementary Information.

## Data Availability

All data generated or analyzed during this study are available from the corresponding author on reasonable request. The three novel *NANS* variants were deposited in a gene-specific database at the Leiden Open Variant Database (LOVD) together with phenotypes: (1) c.207del: variant ID, 0000868160; and DB-ID, NANS_000022 (https://databases.lovd.nl/shared/variants/0000868160); (2) c.607T>C: variant ID, 0000868161; and DB-ID, NANS_000023 (https://databases.lovd.nl/shared/variants/0000868161); and (3) c.133−12T>A: variant ID, 0000868163; and DB-ID, NANS_000021 (https://databases.lovd.nl/shared/variants/0000868163) (the previously reported c.979_981dup:p.(Ile327dup) has already been registered in LOVD).

## References

[1] van Karnebeek CD, Bonafé L, Wen XY, Tarailo-Graovac M, Balzano S, Royer-Bertrand B (2016). NANS-mediated synthesis of sialic acid is required for brain and skeletal development. Nat. Genet..

[2] den Hollander B, Rasing A, Post MA, Klein WM, Oud MM, Brands MM (2021). NANS-CDG: Delineation of the genetic, biochemical, and clinical spectrum. Front. Neurol..

[3] Varki A (2008). Sialic acids in human health and disease. Trends. Mol. Med..

[4] Wang B (2009). Sialic acid is an essential nutrient for brain development and cognition. Annu. Rev. Nutr..

[5] Schnaar RL, Gerardy-Schahn R, Hildebrandt H (2014). Sialic acids in the brain: Gangliosides and polysialic acid in nervous system development, stability, disease, and regeneration. Physiol. Rev..

[6] Roughley PJ, White RJ, Santer V (1981). Comparison of proteoglycans extracted from high and low weight–bearing human articular cartilage, with particular reference to sialic acid content. J. Biol. Chem..

[7] Vincent K, Durrant MC (2013). A structural and functional model for human bone sialoprotein. J. Mol. Graph. Model..

[8] Faye-Petersen OM, Ward K, Carey JC, Knisely AS (1991). Osteochondrodysplasia with rhizomelia, platyspondyly, callosal agenesis, thrombocytopenia, hydrocephalus, and hypertension. Am. J. Med. Genet..

[9] Richards S, Aziz N, Bale S, Bick D, Das S, Gastier-Foster J (2015). Standards and guidelines for the interpretation of sequence variants: A joint consensus recommendation of the American College of Medical Genetics and Genomics and the Association for Molecular Pathology. Genet. Med..

[10] Jaganathan K, Panagiotopoulou SK, McRae JF, Darbandi SF, Knowles D, Li YI (2019). Predicting splicing from primary sequence with deep learning. Cell.

[11] Kuzmiak HA, Maquat LE (2006). Applying nonsense-mediated mRNA decay research to the clinic: Progress and challenges. Trends. Mol. Med..

[12] Matsubara K, Murakami N, Nagai T, Ogata T (2011). Maternal age effect on the development of Prader-Willi syndrome resulting from upd(15)mat through meiosis 1 errors. J. Hum. Genet..

[13] Grondona JM, Pérez-Martín M, Cifuentes M, Pérez J, Jiménez AJ, Pérez-Fígares JM (1996). Ependymal denudation, aqueductal obliteration and hydrocephalus after a single injection of neuraminidase into the lateral ventricle of adult rats. J. Neuropathol. Exp. Neurol..

[14] Izumi R, Niihori T, Suzuki N, Sasahara Y, Rikiishi T, Nishiyama A (2014). GNE myopathy associated with congenital thrombocytopenia: A report of two siblings. Neuromuscul. Disord..

[15] Futterer J, Dalby A, Lowe GC, Johnson B, Simpson MA, Motwani J (2018). Mutation in GNE is associated with a severe form of congenital thrombocytopenia. Blood.

[16] Ma X, Li Y, Kondo Y, Shi H, Han J, Jiang Y (2021). Slc35a1 deficiency causes thrombocytopenia due to impaired megakaryocytopoiesis and excessive platelet clearance in the liver. Haematologica.

[17] Kotzé HF, van Wyk V, Badenhorst PN, Heyns AD, Roodt JP, Lötter MG (1993). Influence of platelet membrane sialic acid and platelet-associated IgG on ageing and sequestration of blood platelets in baboons. Thromb. Haemost..

[18] Schwarzkopf M, Knobeloch K-P, Rohde E, Hinderlich S, Wiechens N, Lucka L (2002). Sialylation is essential for early development in mice. Proc. Natl. Acad. Sci. USA.

[19] Tran C, Turolla L, Ballhausen D, Buros SC, Teav T, Gallart-Ayala H (2021). The fate of orally administered sialic acid: First insights from patients with N-acetylneuraminic acid synthase deficiency and control subjects. Mol. Genet. Metab. Rep..

[20] Mayrhofer M, Viklund B, Isaksson A (2016). Rawcopy: Improved copy number analysis with Affymetrix arrays. Sci. Rep..

[21] Taniuchi K, Miyamoto Y, Uchida Y, Chifu K, Mukai M, Yamaguchi N (1979). A new method for the determination of sialic acid using sialidase combined with NANA-aldolase, and its clinical application. Jpn. J. Clin. Chem..

[22] Shi Y, Xu X, Fang M, Zhang M, Li Y, Gillespie B (2015). Quantitative hydrophilic interaction chromatography–mass spectrometry analysis of N-acetylneuraminic acid and N-acetylmannosamine in human plasma. J. Chromatogr. B..

[23] Wada Y, Okamoto N (2022). Electrospray ionization mass spectrometry of transferrin: Use of quadrupole mass analyzers for congenital disorders of glycosylation. Mass. Spectrom..

[24] Wada Y, Okamoto N (2022). Electrospray ionization mass spectrometry of apolipoprotein CIII to evaluate *O*-glycan site occupancy and sialylation in congenital disorders of glycosylation. Mass. Spectrom..

