# Supplementary material for: Clinical and molecular findings in three Japanese patients with N-acetylneuraminic acid synthetase-congenital disorder of glycosylation (NANS-CDG)
Source: Sci Rep. 2022 Oct 12;12:17079. doi: 10.1038/s41598-022-21751-x (PMC9556533; doi:10.1038/s41598-022-21751-x)
Supplement: Supplementary file 1 — Supplementary Information. [file 41598_2022_21751_MOESM1_ESM.pdf]

SUPPLEMENTARY MATERIALS

Table of Contents

Supplementary Tables 1–4

Supplementary Figure 1

Clinical and molecular findings in three Japanese patients with N-acetylneuraminic acid synthetase-congenital disorder of glycosylation (NANS-CDG)

Supplementary Table 1. Rare variants extracted from whole exome sequencing in patient 1, under the assumption of Mendelian inheritance with complete penetrance in the trio analysis.

	Compound heterozygous variants						*De novo* variants	Hemizygous variant				
Gene (Chromosome)	*NANS* (Chr. 9)	*NANS* (Chr. 9)	*KIF21A* (Chr. 12)	*KIF21A* (Chr. 12)	*CD68* (Chr. 17)	*CD68* (Chr. 17)	*ZNF624* (Chr. 17)	*P2RY10* (Chr. X)	*TSC22D3* (Chr. X)	*CCDC160* (Chr. X)	*ADGRG4* (Chr. X)	*NSDHL* (Chr. X)
Variant (GenBank)	NM_018946.4	NM_018946.4	NM_001173464.2	NM_001173464.2	NM_001251.3	NM_001251.3	NM_020787.4	NM_014499.4	NM_001318468.1	NM_001101357.3	NM_153834.4	NM_015922.3
	c.207del	c.979_981dup	c.1854_1862del	c.1886G>T	c.229G>A	c.589A>G	c.263G>A	c.32T>A	c.265A>G	c.157A>G	c.426A>G	c.1108C>T
	p.(Arg69Serfs*57)	p.(Ile327dup)	p.(Glu619_Glu621del)	p.(Ser629Ile)	p.(Ala77Thr)	p.(Asn197Asp)	p.(Arg88Lys)	p.(Phe11Tyr)	p.(Ile89Val)	p.(Arg53Gly)	p.(Ile142Met)	p.(Arg370Trp)
Inheritance	Maternal	Paternal	Maternal	Paternal	Maternal	Paternal	*De novo*	Maternal	Maternal	Maternal	Maternal	Maternal
<Frequency>												
gnomeAD East Asia	0.0000	0.0000	0.0000	0.0015	0.0000	0.0000	0.0000	0.0004	0.0038	0.0016	0.0003	0.0011
HGVD	0.0000	0.0000	0.0000	0.0041	0.0004	0.0017	0.0000	0.0000	0.0019	0.0067	0.0000	0.0039
14KJPN	0.0000	0.0003	0.0000	0.0067	0.0000	0.0027	0.0000	0.0081	0.0022	0.0042	0.0011	0.0011
In-house (n = 218)	0.0000	0.0000	0.0000	0.0069	0.0000	0.0023	0.0000	0.0092	0.0000	0.0069	0.0000	0.0000
<Pathogenicity>												
CADD PHRED	Not predicted	Not predicted	Not predicted	1% most deleterious	Deleterious	1% most deleterious	Deleterious	Deleterious	1% most deleterious	Non-deleterious	Deleterious	1% most deleterious
Score				24.5	10.3	23.8	18.1	15.5	23.6	5.7	17.8	23.9
PolyPhen-2 HumVar	Not predicted	Not predicted	Not predicted	Probably damaging	Benign	Probably damaging	Benign	Benign	Possibility damaging	Benign	Probably damaging	Possibility damaging
Score				0.991	0.017	0.998	0.005	0.064	0.894	0.007	0.948	0.796
SIFT	Not predicted	Not predicted	Not predicted	Damaging	Tolerated	Tolerated	Tolerated	Damaging	Tolerated	Tolerated	Damaging	Damaging
Score				0.003	0.346	0.779	0.195	0.037	0.243	0.112	0.000	0.001
MutationTaster	Disease causing	Disease causing	Disease causing	Disease causing	Polymorphism	Disease causing	Polymorphism	Polymorphism	Disease causing	Polymorphism	Polymorphism	Disease causing
Score	1.000	0.929	1.000	0.999	1.000	0.567	0.997	0.995	1.000	1.000	1.000	0.999
<Phenotype>												
OMIM	Spondyloepimetaphyseal dysplasia, Camera-Genevieve type		Fibrosis of extraocular muscles		Unknown		Unknown	Unknown	Unknown	Unknown	Unknown	CHILD syndrome

Shown are non-synonymous rare variants with minor allele frequencies of ≤ 0.01 in all the public and in-house databases employed.

The URLs utilized are as follows; *in silico* analyses have been performed using default parameters.

1) GenBank: https://www.ncbi.nlm.nih.gov/genbank.

2) gnomAD (Genome Aggregation Database): http://gnomad.broadinstitute.org/.

3) HGVD (Human Genetic Variation Database): http://www.hgvd.genome.med.kyoto-u.ac.jp/.

4) 14KJPN (Whole-genome sequences of 14,000 healthy Japanese individuals and construction of the highly accurate Japanese population reference panel): https://ijgvd.megabank.tohoku.ac.jp/.

5) CADD (Combined Annotation–Dependent Depletion): http://cadd.gs.washington.edu/ (Current version: 1.6, GRCh37/hg19); PHRED scores of > 10–20 are regarded as deleterious, and those of > 20 indicates the 1% most deleterious.

6) Polyphen-2 Hum Var: http://genetics.bwh.harvard.edu/pph2/ (Current version: 2.2.2, GRCh37/hg19); HumVar scores were evaluated as 0.000 (most probably benign) to 1.000 (most probably damaging).

7) SIFT (Sorting Intolerant From Tolerant): http://sift.jcvi.org/ (Current version: Mar 2013; GRCh37/Ensembl 66); Scores of ≤0.05 and those > 0.05 are assessed as damaging and tolerated, respectively.

8) MutationTaster: http://www.mutationtaster.org/ (MutationTaster2, GRCh37/Ensembl 69); Alterations are classified as disease causing or polymorphisms, and the high scores of ~1.00 indicate the high probability of disease-causing variant or polymorphism.

9) OMIM: https://www.ncbi.nlm.nih.gov/omim.

Supplementary Table 2. Rare variants extracted from whole exome sequencing in patient 2, under the assumption of Mendelian inheritance with complete penetrance in the trio analysis.

	Homozygous variant				*De novo* variants	Compound heterozygous variants	
Gene (Chromosome)	*NANS* (Chr. 9)	*CD274* (Chr. 9)	*FBP1* (Chr. 9)	*OR13C8* (Chr. 9)	*IFITM10* (Chr. 11)	*DNAH11* (Chr. 7)	*DNAH11* (Chr. 7)
Variant (GenBank)	NM_018946.4	NM_014143.4	NM_000507.4	NM_001004483.1	NM_001170820.4	NM_001277115.2	NM_001277115.2
	c.979_981dup	c.791-4C>G	c.405C>T	c.274G>C	c.514A>G	c.7012C>T	c.5491T>A
	p.(Ile327dup)		p.(=)	p.(Val92Leu)	p.(Ile172Val)	p.(Arg2338Trp)	p.(Ser1831Thr)
Inheritance	Maternal	Maternal	Maternal	Maternal	*De novo*	Maternal	Paternal
<Frequency>							
gnomeAD East Asia	0.0000	0.0004	0.0003	0.0027	0.0000	0.0001	0.0000
HGVD	0.0000	0.0056	0.0032	0.0061	0.0000	0.0005	0.0000
14KJPN	0.0003	0.0053	0.0044	0.0077	0.0000	0.0000	0.0001
In-house (n = 218)	0.0000	0.0023	0.0023	0.0069	0.0000	0.0000	0.0000
<Pathogenicity>							
CADD PHRED	Not predicted	Non-deleterious	Deleterious	Deleterious	1% most deleterious	1% most deleterious	1% most deleterious
Score		1.6	12.8	12.4	24.0	34.0	27.4
PolyPhen-2 HumVar	Not predicted	Not predicted	Not predicted	Benign	Not predicted	Benign	Benign
Score				0.003		0.296	0.341
SIFT	Not predicted	Not predicted	Not predicted	Damaging	Tolerated	Damaging	Damaging
Score				0.001	0.115	0.004	0.003
MutationTaster	Disease causing	Not predicted	Disease causing	Polymorphism	Disease causing	Disease causing	Disease causing
Score	0.929		1.000	0.870	0.639	0.948	0.544
<Phenotype>							
OMIM	Spondyloepimetaphyseal dysplasia, Camera-Genevieve type	Unknown	Fructose-1,6-bisphosphatase deficiency	Unknown	Unknown		Ciliary dyskinesia, primary

Shown are non-synonymous rare variants with minor allele frequencies of ≤ 0.01 in all the public and in-house databases employed.

The URLs utilized are shown in the footnotes of Supplementary Table 1; *in silico* analyses have been performed using default parameters.

Supplementary Table 3. Rare variants extracted from whole exome sequencing in patient 3, under the assumption of Mendelian inheritance with complete penetrance in the trio analysis.

	Compound heterozygous variants				*De novo* variants		Hemizygous variant				
Gene (Chromosome)	*NANS* (Chr. 9)	*NANS* (Chr. 9)	*PKD1L2* (Chr. 16)	*PKD1L2* (Chr. 16)	*CFAP74* (Chr. 1)	*HACL1* (Chr. 3)	*ZNF41* (Chr. X)	*WDR45* (Chr. X)	*ERCC6L* (Chr. X)	*PCDH19* (Chr. X)	*PASD1* (Chr. X)
Variant (GenBank)	NM_018946.4	NM_018946.4	NM_001076780.2	NM_001076780.2	NM_001304360.2	NM_012260.4	NM_001324140.2	NM_007075.4	NM_017669.4	NM_001184880.2	NM_173493.3
	c.607T>C	c.133-12T>A	c.1658C>T	c.1906C>T	c.1860C>T	c.679G>C	c.1301C>T	c.841G>A	c.2801G>C	c.2250C>A	c.1874A>T
	p.(Tyr203His)		p.(Ala553Val)	p.(Arg636Cys)	p.(=)	p.(Ala227Pro)	p.(Thr434Met)	p.(Val281Met)	p.(Ser934Thr)	p.(Asp750Glu)	p.(Tyr625Phe)
Inheritance	Maternal	Paternal	Maternal	Paternal	*De novo*	*De novo*	Maternal	Maternal	Maternal	Maternal	Maternal
<Frequency>											
gnomeAD East Asia	0.0000	0.0000	0.0045	0.0000	0.0000	0.0000	0.0002	0.0041	0.0002	0.0002	0.0003
HGVD	0.0005	0.0000	0.0093	0.0038	0.0000	0.0000	0.0005	0.0000	0.0000	0.0000	0.0050
14KJPN	0.0000	0.0003	0.0074	0.0017	0.0000	0.0000	0.0003	0.0010	0.0002	0.0002	0.0052
In-house (n = 218)	0.0000	0.0000	0.0046	0.0023	0.0000	0.0000	0.0000	0.0023	0.0000	0.0046	0.0000
<Pathogenicity>											
CADD PHRED	1% most deleterious	Not predicted	Deleterious	Deleterious	Deleterious	1% most deleterious	1% most deleterious	1% most deleterious	Non-deleterious	Non-deleterious	Deleterious
Score	24.2		13.9	16.7	13.5	20.9	27.5	22.2	0.3	4.8	10.6
PolyPhen-2 HumVar	Probably damaging	Not predicted	Benign	Possibility damaging	Not predicted	Probably damaging	Probably damaging	Possibility damaging	Probably damaging	Probably damaging	Probably damaging
Score	0.950		0.000	0.804		0.918	0.998	0.018	0.000	0.017	0.207
SIFT	Damaging	Not predicted	Not predicted	Not predicted	Not predicted	Damaging	Damaging	Tolerated	Tolerated	Tolerated	Damaging
Score	0.000					0.003	0.000	0.607	0.874	0.220	0.000
MutationTaster	Disease causing	Not predicted	Polymorphism	Polymorphism	Disease causing	Polymorphism	Disease causing	Polymorphism	Polymorphism	Polymorphism	Polymorphism
Score	1.000		1.000	0.999	1.000	1.000	0.863	0.000	1.000	0.912	1.000
<Phenotype>											
OMIM	Spondyloepimetaphyseal dysplasia, Camera-Genevieve type		Unknown		Unknown	Unknown	Unknown	Neurodegeneration with brain iron accumulation 5	Unknown	Developmental and epileptic encephalopathy 9	Unknown

Shown are non-synonymous rare variants with minor allele frequencies of ≤ 0.01 in all the public and in-house databases employed.

The URLs utilized are shown in the footnotes of Supplementary Table 1; *in silico* analyses have been performed using default parameters.

Supplemental Table 4. Primers used in this study.

Sequence analysis using gDNA	Forward	Reverse
c.133-12T>A & c.207delG	TGGGTGAAAGAGCGAAACTC	GGGAACAGAAAGGATGAGCA
c.607T>C	GGCTCAACCAGATACGCTTC	CTTCTCATTGCAGGCCATCT
c.979_981dup	AGCCCTTTCCTTTGCACTTT	ACAGCATCAGCAAGGGAACT
Sequence and qPCR analyses using cDNA	Forward	Reverse
c.133-12T>A	CCGGACCCAGACTGGTAGT (P1)	ACTGGTCATGGCTGAACTCC (P2)
c.607T>C	TCCAGTGGGATGCAGTCAAT	CTTCTCATTGCAGGCCATCT

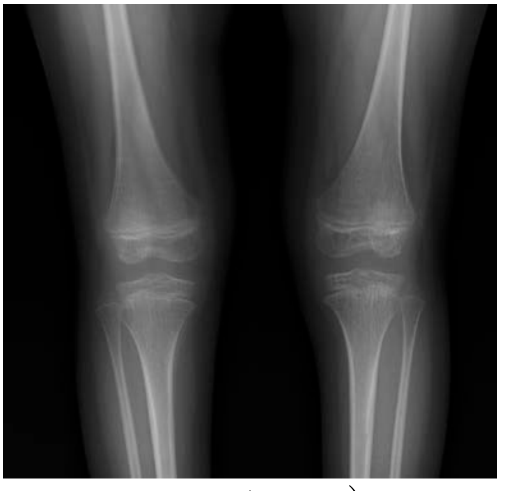

Patient 2 (7 y 3 m）

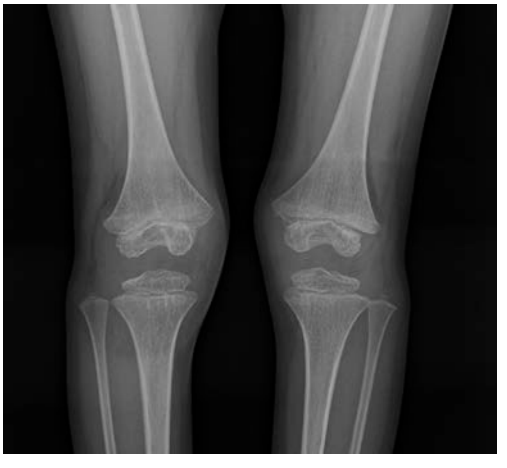

Patient 3 (5 y 10 m)

Supplementary Figure 1. Roentgenograms showing longitudinal striations. Epiphyseal findings of patient 3 appear sclerotic and irregular (flocky).
